# Excitatory‐inhibitory modulation of transcranial focus ultrasound stimulation on human motor cortex

**DOI:** 10.1111/cns.14303

**Published:** 2023-06-12

**Authors:** Tingting Zhang, Bingqi Guo, Zhentao Zuo, Xiaojing Long, Shimin Hu, Siran Li, Xin Su, Yuping Wang, Chunyan Liu

**Affiliations:** ^1^ Department of Neurology, Xuanwu Hospital Capital Medical University Beijing China; ^2^ Beijing Key Laboratory of Neuromodulation Beijing China; ^3^ State Key Laboratory of Brain and Cognitive Science, Beijing MR Center for Brain Research, Institute of Biophysics Chinese Academy of Sciences Beijing China; ^4^ Hefei Comprehensive National Science Center Institute of Artificial Intelligence Hefei China; ^5^ Sino‐Danish College University of Chinese Academy of Sciences Beijing China; ^6^ Shenzhen Institutes of Advanced Technology Chinese Academy of Sciences Shenzhen China; ^7^ Institute of Sleep and Consciousness Disorders, Center of Epilepsy, Beijing Institute for Brain Disorders Capital Medical University Beijing China; ^8^ Hebei Hospital of Xuanwu Hospital Capital Medical University Shijiazhuang China; ^9^ Neuromedical Technology Innovation Center of Hebei Province Shijiazhuang China

**Keywords:** cortical plasticity, proton magnetic resonance spectroscopy, transcranial focus ultrasound stimulation, transcranial magnetic stimulation

## Abstract

**Aims:**

Transcranial focus ultrasound stimulation (tFUS) is a promising non‐invasive neuromodulation technology. This study aimed to evaluate the modulatory effects of tFUS on human motor cortex (M1) excitability and explore the mechanism of neurotransmitter‐related intracortical circuitry and plasticity.

**Methods:**

Single pulse transcranial magnetic stimulation (TMS)‐eliciting motor‐evoked potentials (MEPs) were used to assessed M1 excitability in 10 subjects. Paired‐pulse TMS was used to measure the effects of tFUS on GABA‐ and glutamate‐related intracortical excitability and ^1^H‐MRS was used to assess the effects of repetitive tFUS on GABA and Glx (glutamine + glutamate) neurometabolic concentrations in the targeting region in nine subjects.

**Results:**

The etFUS significantly increased M1 excitability, decreased short interval intracortical inhibition (SICI) and long interval intracortical inhibition (LICI). The itFUS significantly suppressed M1 excitability, increased SICI, LICI, and decreased intracortical facilitation (ICF). Seven times of etFUS decreased the GABA concentration (6.32%), increased the Glx concentration (12.40%), and decreased the GABA/Glx ratio measured by MRS, while itFUS increased the GABA concentration (18.59%), decreased Glx concentration (0.35%), and significantly increased GABA/Glx ratio.

**Conclusion:**

The findings support that tFUS with different parameters can exert excitatory and inhibitory neuromodulatory effects on the human motor cortex. We provide novel insights that tFUS change cortical excitability and plasticity by regulating excitatory‐inhibition balance related to the GABAergic and glutamatergic receptor function and neurotransmitter metabolic level.

## Graphical abstract

MRI‐guided tFUS with two sets of different parameters produces excitatory and inhibitory neuro‐modulatory effects on the human motor cortex. The potential mechanism may be to regulate the GABAergic and glutamatergic receptor function and neurotransmitter metabolic level.

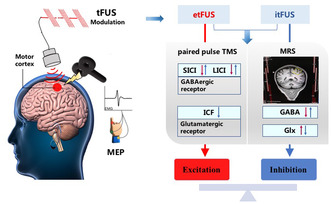

## 1 INTRODUCTION

Neuromodulation technologies are important therapeutic modalities for neurological diseases including dementia, Parkinson's disease, epilepsy, depression.^1^, ^2^ However, the modulation effects of noninvasive strategies, such as transcranial direct current stimulation (tDCS) and repetitive transcranial magnetic stimulation (rTMS) are limited to the cortical surface and less well‐focused.^3^, ^4^ The invasive deep brain stimulation (DBS) can target deep brain structure exactly, but induce risks associated with brain surgery.^4^, ^5^ Low intensity transcranial focused ultrasound stimulation (tFUS) is an emerging non‐invasive neuromodulation technology in which an ultrasound beam is focused onto specific brain areas, with the advantages of high spatial resolution, targeting deep brain regions and better safety.^6^

Research on the potential clinical value of focused ultrasound as a neuromodulation method started half a century ago, with interest increasing dramatically over the past decade.^7^ Studies have shown that tFUS modulates neural activity in the human motor cortex,^8^, ^9^ somatosensory cortex,^10,11^ right inferior frontal gyrus,^12^ thalamus^13^ and visual cortex.^13^, ^14^ Several studies have reported beneficial effects of tFUS applied to patients with chronic pain, posttraumatic disorder of consciousness, and Alzheimer's disease.^15^, ^16^, ^17^ Sanguinetti et al.^11^ reported that tFUS targeting the right prefrontal cortex enhances mood and changed the functional connectivity related to emotional regulation networks.

Despite this growing corpus, the current knowledge on the precise mechanisms of how tFUS modulate the neural activity and plasticity is limited. Investigations suggest that ultrasound primarily exerts its modulatory effects through mechanical action on cell membranes, notably affecting ion channel gating.^18^ The study of Zhang et al.^19^ reported that FUS reduced the network connections of epilepsy circuits and change the structure of the brain network at the whole‐brain level. It has been reported that ultrasound exposure in anesthetized rats modulated the extracellular serotonin, dopamine and GABA levels, as well as neurotrophic factors.^20^, ^21^, ^22^ These findings highlight that tFUS can not only transiently alter neuronal activity through regulating spiking, but also produce longer lasting effects that affect the global connectivity possibly through modulating synaptic function.

Balanced excitatory and inhibitory activity (i.e., E/I balance) is a canonical feature in models of healthy brain function. TMS is a noninvasive method to measure cortical excitability by applying magnetic pulses to the brain and measuring the resulting motor response. Additionally, TMS applied in a paired‐pulse sequence may provide insights into the function of cortical inhibitory and excitatory interneurons depending on the interval between two stimuli. Short interval intracortical inhibition (SICI) with short interstimulus intervals (ISIs) of 1–5 ms is believed to be the product of axonal refractoriness and low threshold GABA_A_ receptor‐mediated inhibition. Long interval intracortical inhibition (LICI) with ISIs of 50–400 ms is related to the function of GABA_B_ receptors. Intracortical facilitation (ICF) with ISIs of 6–30 ms is thought to be mediated by glutamatergic N‐methyl‐D‐aspartate (NMDA) receptors.^23^, ^24^ Proton magnetic resonance spectroscopy (MRS), which can quantify various metabolites by distinguishing molecular properties, is the only way to non‐invasively assess concentrations of primary excitatory (glutamate) and inhibitory (γ‐aminobutyric acid, GABA) neurotransmitters in human brain.^25^ The combination of electrophysiology and MRS may reveal complementary and comprehensive information on glutamatergic and GABAergic neurotransmission.^26^

In this study, we hypothesize that the tFUS modulates the cortical excitability patterns by affecting plasticity related to GABAnergic and glutamatergic excitation and inhibition balance. We aimed to explore the hypothetical mechanism through TMS and MRS methods with two sets of different parameters of tFUS targeting the primary motor cortex (M1) of healthy human participants. To our knowledge, we conducted the first MRS measurement to evaluate how repeated application of tFUS affects the concentration of GABA and glutamine in human cortex, and this is the first study to explore the neural regulatory mechanism of tFUS by combining MRS and TMS technology.

## 2 EXPERIMENTAL PROCEDURES

### 2.1 Overview of experimental procedures

We recruited 12 healthy volunteers under the approval of the ethics committee of the Xuanwu Hospital, Capital Medical University. One subject withdrew due to illness, and one withdrew due to failure of MEP detection. The remaining 10 subjects completed Experiment 1 (four males and six females, age = 27 ± 5.8). One of the 10 subjects withdrew for personal reasons, and nine completed Experiment 2 and Experiment 3 (four males and five females, age = 27.5 ± 7.1). All participants provided written informed consent prior to enrollment and received monetary compensation upon completion. The participants were selected after pass the following inclusion criteria: naïve to stimulation; right‐handed; no neurological or other serious medical issues; no metallic implants, such as pacemakers, DBS treatment devices, or cardiac stents; no current pregnancy; no drug or alcohol addiction; no participation in another study within the last 12 weeks; no substance intake that affect cortical excitability (drugs, coffee, tea, etc) and no sleep deprivation 1 week before and during the study.

**FIGURE 1:**
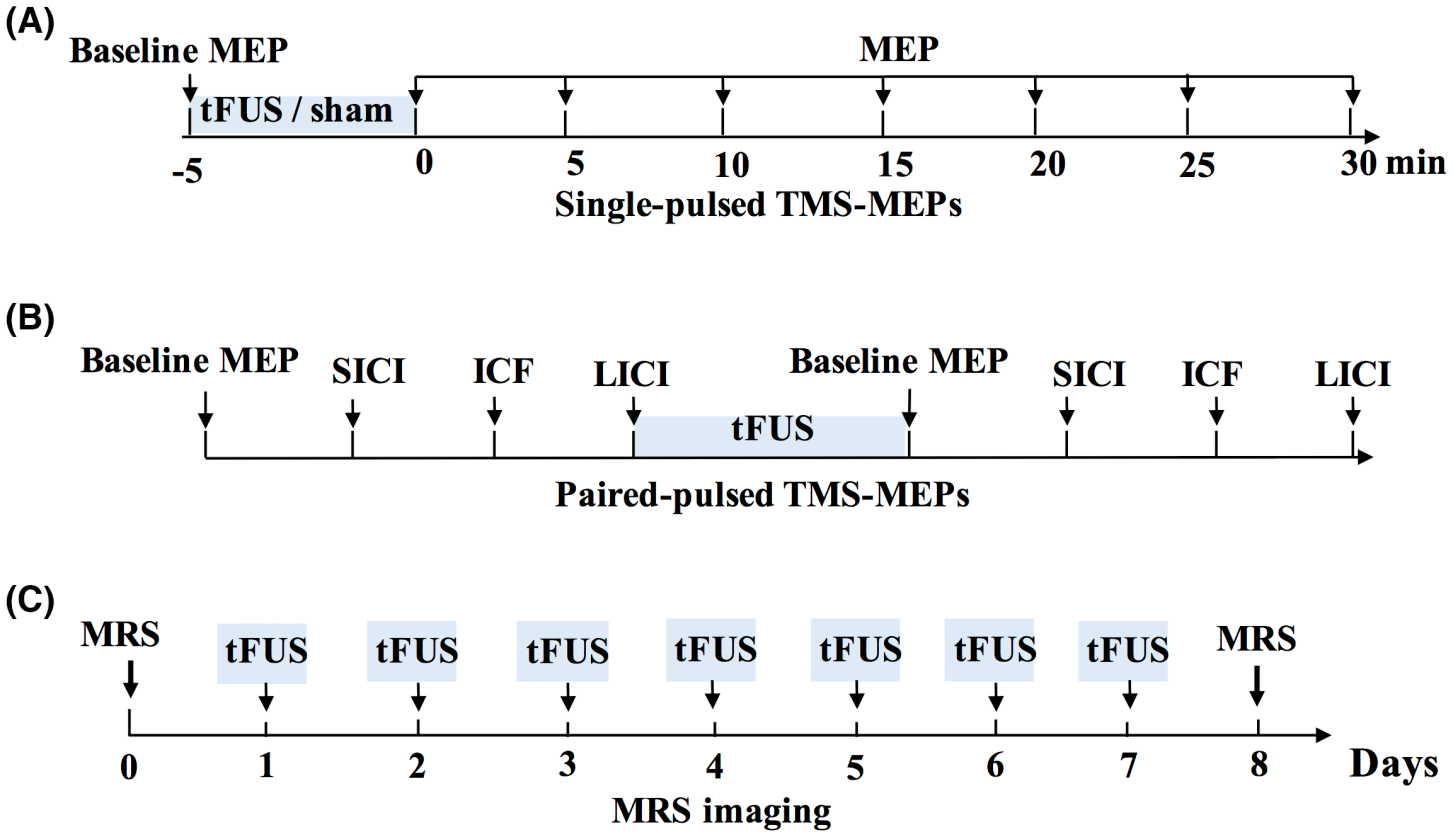
Experimental paradigms.

Three experiments were conducted (Figure 1A–C). Experiment 1: Effect of the tFUS on the excitability of motor cortex by measuring single pulsed TMS elicited MEP amplitudes. Sham, etFUS, and itFUS stimulation were applied for 5 min and MEPs were tested before and after stimulation. Subjects received excitatory and inhibitory and sham stimuli 1 week apart. The order of the excitatory and inhibitory and sham tFUS is random. Experiment 2: Effects of tFUS on paired‐pulse TMS induced intracortical inhibition and facilitation. The etFUS and itFUS were applied for 5 min and SICI, ICF, and LICI were tested before and after stimulation. Subjects received both excitatory and inhibitory stimuli 1 week apart. The order of the excitatory and inhibitory tFUS is random. Experiment 3: Effect of repeated tFUS on the GABA and glutamine levels in the target region measured by ^1^H‐MRS before and after the stimulation. We used the excel functions to randomly divide the nine subjects into two groups to receive etFUS or itFUS stimuli 5 min once a day for 7 consecutive days. Before sonication stimulation, magnetic resonance imaging (MRI) scans were conducted across all the participants for the preparation of image‐guided application of tFUS. Data of the single‐pulse TMS test were obtained from the 10 healthy participants. Nine volunteers completed the paired‐pulse TMS test and MRS imaging scan.

### 2.2 tFUS waveform and quantitative acoustic field mapping

Transcranial ultrasonic waveforms were generated using an Ultrasound Neurostimulation System (GreenValley BrainTech Medical Techology Corportion). Briefly, channel output was set to deliver a signal to drive the custom‐designed focused ultrasound transducer having a center frequency of 0.5 MHz, a diameter of 48 mm and a focal length of about 30 mm (the distance from sound passing membrane to focal point). We measured the acoustic pressure profile of the waveform using a calibrated hydrophone (NH1000, PA Corporation) whose signal was amplified by a PA18081 preamplifier (PA Corporation). The hydrophone, ultrasound transducer and skull fragment were positioned within an organic glass water tank, and the hydrophone was mounted on a three‐axis stage. Commercial software written in LabVIEW (National Instruments) was used to control the three‐axis stage as well as recording of the corresponding wave‐form as measured by the hydrophone. To test the effects of a human skull on FUS fields, we inserted a human parietal bone (rehydrated for 48 h) between the transducer and the hydrophone, the thickness of skull at the test area is approximately 5 mm. Scans around the axis (*z* axis) were first performed to find the focal distance; next, a 12.3 × 12.3 mm scan was performed at this distance to obtain an *x*‐*y* acoustic pressure map at the focal plane and additional 20.5 × 50.5 mm scan was implemented to obtain an *x‐z* acoustic pressure map at a plane which containing the peak acoustic pressure point. The transducer is fitted with a waterish acoustic collimator which equipped with a sound passing membrane. In the operation of sound field test, a roughly estimate of the distance between membrane and the outer surface of the skull was 2 mm (scalp thickness was replaced by water). An acoustic simulation FEA model of ultrasound penetrating the skull was created using Onscale (projection of tFUS fields into a realistic head model). Briefly, skull was segmented from CT images and two‐dimensional FEM model of the head was created. It is well known that the acoustic impedance of many kinds of human tissue is similar to water, hence, the scalp, brain tissue, and cerebrospinal fluid were replaced by water.

### 2.3 Ultrasound stimulation targeting the motor cortex

**FIGURE 2:**
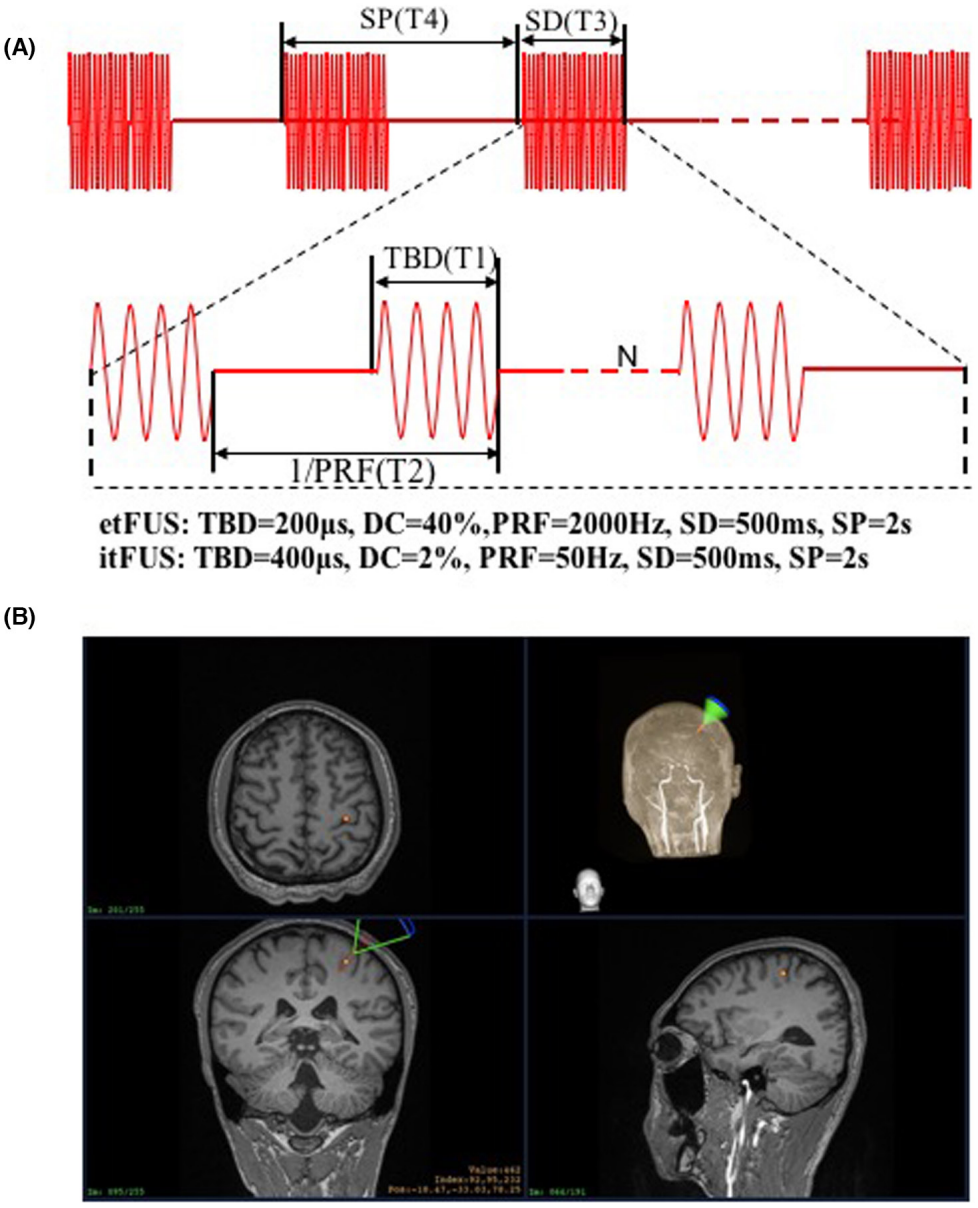
(A) The pulse parameters of tFUS. (B) MRI image guided tFUS focusing over left M1. PRF, pulse repetition frequency; SD, sonication duration; SP, Stimulus Period; TBD, tone‐burst duration.

The ultrasound pulse mode is determined by four elements displayed on the console: pulse width (T1), pulse repetition period (T2), burst duration (T3), burst period (T4). The pulses have an associated pulse repetition frequency (PRF, the rate of the pulses delivered) and are repeated at this frequency for a length of time defined by T2. The duty cycle (DC) is the proportion of each pulse filled with ultrasound cycles (T1/T2 as a percentage value). T4 includes burst duration and burst interval. As shown in Figure 2A, we used two sets of parameters: (1) etFUS which displayed excitatory effects: T1 = 200 μs, T2 = 0.5 ms, T3 = 500 ms, T4 = 2 s, PRF = 2000 Hz, DC = 40%. (2) itFUS which displayed inhibitory effects: T1 = 400 μs, T2 = 20 ms, T3 = 500 ms, T4 = 2 s, PRF = 50 Hz, DC = 2%. For MEP tests, Parameter sets of etFUS and itFUS were randomly assigned to five participants respectively for the sham stimulation.^27^, ^28^, ^29^ Every participant shall participate in three tests (etFUS, itFUS and sham) at least 1 week apart in random order.

Focus ultrasound stimulation navigation and guidance: The subjects sat comfortably in a chair without any restrictions on their head. They were not allowed to move their head during the registration and the FUS treatment period. T1‐weighted MPRAGE (TR = 2530 ms, TE = 2.98 ms, TI = 1100 ms, Voxel size = 1.0 × 1.0 × 1.0 mm^3^) MRI was acquired to get the anatomical information for each participant. The acquired MRI data were loaded in infrared image‐guided FUS navigation software built‐in system. The stimulation target area (hand motor cortex) was marked according to the anatomical MRI image (Figure 2B, yellow dot). The focus position (Figure 2B, red spindle) and the position of the FUS path (Figure 2B, green sector) relative to the target point are displayed and updated in real time on the monitor. The operator manually adjusts the position and spatial direction of the transducer to superposing the FUS focus on the target area. The incident sound beam should be as perpendicular to the curvature of the skull as possible, and the ultrasonic gel should be filled in advance to eliminate the air between the transducer and the scalp (Figure 2B).

### 2.4 Measurement of motor‐cortical excitability

Motor cortex excitability was assessed by measuring peak‐to‐peak MEP amplitudes potentials (MEPs) elicited by TMS applied over left M1 in the first dorsal interosseous (FDI) of the dominant right hand. For all TMS procedures, a Magstim Rapid^2^ stimulator (Magstim Company Ltd.) was used and a 70‐mm figure‐of‐eight coil was placed over the motor cortex. The handle of the coil was oriented posterior to the midline at a 45° angle, such that the electromagnetic currents flowed perpendicular to the central sulcus. The optimal scalp location (hot‐spot) for the FDI stimulation was determined using TMS by moving the coil over the scalp in 1‐cm steps around a spot 1 cm anterior to the C3 site of the 10–20 EEG Electrode Placement Method. The hotspot was then marked on the skull with a waterproof pen to ensure reliable TMS coil repositioning during the experiments. MEPs were identified in electromyography (EMG) data collected through Ag–AgCl surface electrodes placed over the muscle using the belly‐tendon montage. The EMG activity was amplified (1000×), filtered (10 Hz to 3 kHz), and subsequently sampled at 4 kHz (ISA1008 EP, Micromed, ITA). Data were stored on a computer for offline analysis.

Participants were comfortably seated in a chair to remain relaxed throughout the experiment. Resting motor threshold (RMT) of each participant was determined according to international guidelines as the stimulator's output able to elicit reproducible MEPs (at least 50 μV in amplitude) in 5 of 10 subsequent trials in a muscle at rest.^30^ Cortical excitability was probed by measuring peak‐to‐peak amplitudes of MEPs elicited by TMS pulses with an intensity of 120% of RMT. Ten MEPs were recorded at baseline (before ultrasonic stimulation delivery), immediately after the completion of the tFUS or sham stimulation, and every 5 min thereafter (up to 30 min). A total of seven times following the ultrasonic stimulation were evaluated. The TMS pulses were delivered with an average inter‐stimulus interval of 8 s that varied randomly by ±1 s. (Figure 1A).

### 2.5 Paired‐pulse TMS test

After seated in a chair and hooked up to EMG, subjects underwent the following procedures: (1) RMT measurement as before, then the average of the first 10 MEP with 120% of RMT as stimulus intensity was obtained as the baseline MEP; (2) SICI: paired (conditioning/test) pulse at an interstimulus interval (ISI) of 2 and 5 ms; (3) ICF: paired (conditioning/test) pulse at an ISI of 10 and 15 ms; (4) LICI: paired (conditioning/test) pulse at an ISI of 100 and 150 ms; (5) 5 min of tFUS with the same parameters used before (etFUS and itFUS at least 1 week apart in random order); (6) Repeat (1)–(4) above immediately after tFUS. If RMT was changed after intervention, this new threshold was utilized for MEP measurements post stimulation. The conditioning stimulus (CS) was set to a sub‐threshold intensity of 80% RMT, the test stimulus (TS) was set to 120% RMT for both SICI and ICF. Supra‐threshold intensity of 120% RMT was used for both the CS and TS for LICI.^31^ (Figure 1B).

### 2.6 MRS acquisition and data analysis

The MRS acquisition was performed before and after 7 days of tFUS (Figure 1C). All measurements were performed with a 3.0 T MR scanner (Skyra, Siemens Healthineers). Each experimental session started with the acquisition of sagittal T1‐weighted images (same as above), which were used to carefully place a 25 × 30 × 30 mm^3^ voxel of interest (VOI) within the hand area of the left M1(same as the target of tFUS). The MEGA‐PRESS sequence was used for GABA editing, with the parameters as follows: TR = 2000 ms, TE = 68 ms, averages = 96, bandwidth = 2000 Hz, editing pulse BW = 62.10 Hz. J‐evolution for GABA was refocused during odd‐numbered acquisitions (ON) but not during even‐numbered acquisitions (OFF) by applying Gaussian inversion pulse to the ^3^CH2 resonance of GABA at 1.9 ppm (ON) and symmetrically about the water peak at 7.5 ppm (OFF), respectively. Water suppression was carried out using chemical shift‐selective (CHESS) pulses after automatic optimization. FASTMAP shimming of the VOI was conducted automatically before each acquisition. The difference of the “ON” and “OFF” spectra provided an edited spectrum of GABA.

Quantification was performed using the Gannet 2.0 toolkit, a Matlab‐based quantitative batch analysis tool for analyzing GABA MEGA‐PRESS spectra.^32^ Gannet contains two modules: GannetLoad and GannetFit. The GannetLoad module is used to parse certain variables from the data headers, apply a line broadening of 3 Hz, and frequency and phase correct the individual spectra using Spectral Registration.^33^ GannetFit uses a single‐Gaussian model to fit the edited GABA signal. GABA and Glx were quantified relative to water, as a concentration in institutional units (i.u.). Only spectra with a relative fitting error (FitError) of GABA generated by Gannet smaller than 15% were enrolled in the final statistical analysis. The fitting errors and signal‐to‐noise ratios (SNR) of GABA signals were also recorded.

### 2.7 Statistical analysis

The statistical analysis was performed using GraphPad Prism 8.0. The statistical significance was set at *p* < 0.05 and group data are present as mean ± SEM. Shapiro–Wilk test was conducted to test the normality and revealed normal distribution for all continuous variables. In MEP test, the result was subtracted by 100% (baseline is 100%) to obtain the ΔMEPs percentage. The MEP modulation in percentage was analyzed by two‐factor (Time and Stimulation), mixed‐design ANOVA. The sphericity of the set of variables was assessed by the Mauchly test and, when it was violated, the Greenhouse–Geisser correction was used. The effect size of mixed‐design ANOVA was determined using partial eta squared (ηp^2^). The ΔMEPs percentage of etFUS stimulation group and itFUS stimulation group were compared with those of the sham group at the time points of 0, 5, 10, 15, 20, 25, 30 min, respectively. Adjusted *p* value < 0.0071 (0.05/7) between‐group were considered to be statistically significant after Bonferroni correction. Differences of SICI, ICF, LICI as well as the levels of neurometabolites (GABA, Glx, GABA/Glx) before and after tFUS treatment were analyzed using paired *t*‐tests.

## 3 RESULTS

### 3.1 Acoustic beam properties of focused ultrasound

**FIGURE 3:**
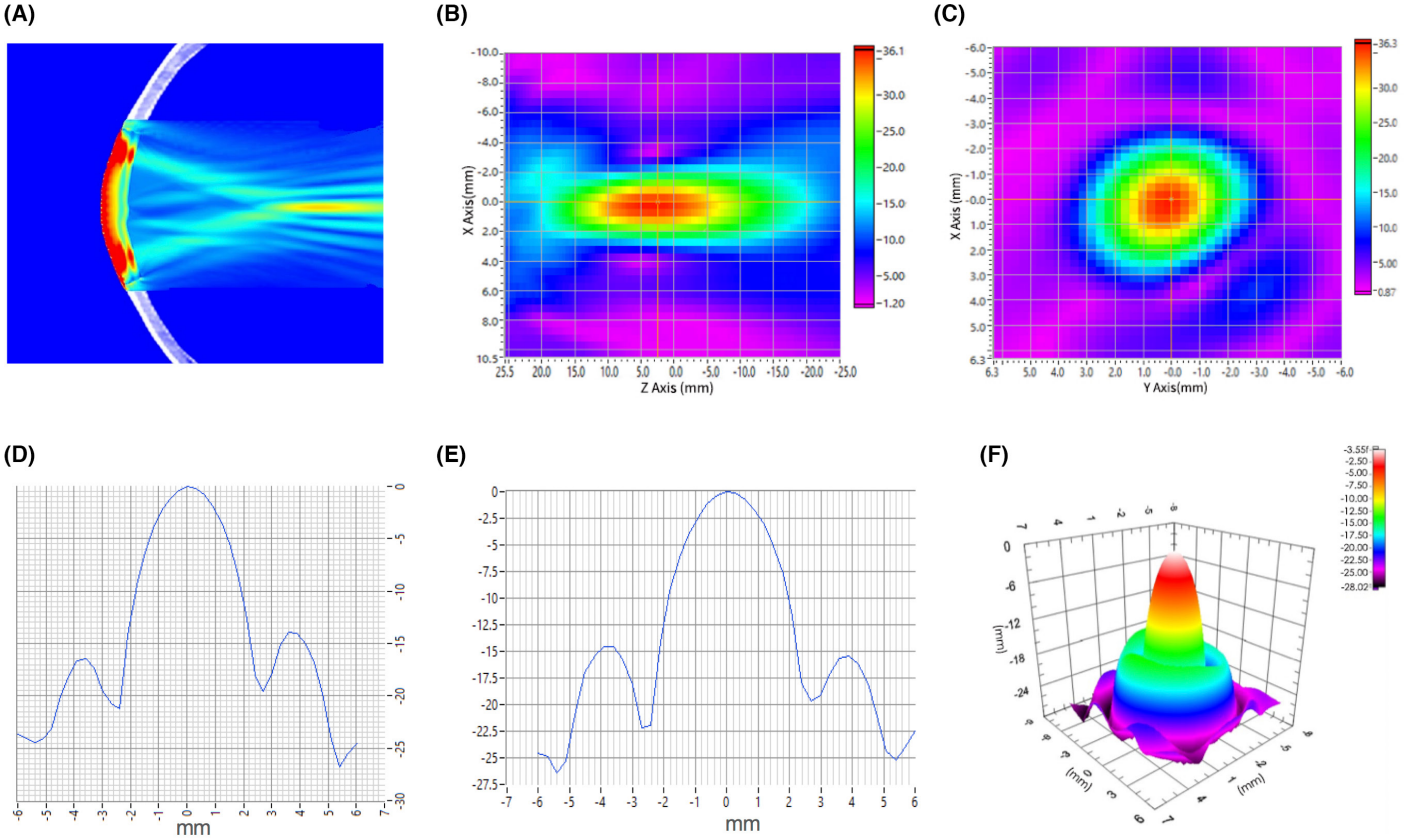
Acoustic intensity fields after transcranial transmission through hydrated human skull bone. (A) Simulation of ultrasound field through human skull bone. (B) Cross section through the focus center in the direction of parallel acoustic beam emission. (C) Cross section through the focus center perpendicular to the direction of acoustic beam emission. (D) Line plots illustrate the lateral (*x*) and (E) vertical (*y*) peak normalized acoustic intensity profiles for the acoustic beam in the focal plane. (F) The Focal plane sound pressure distribution.

Using a calibrated hydrophone mounted on a motorized, three‐axis stage, we recorded acoustic pressure fields transmitted from the FUS transducer through human cranium (Figure 3A). The longitudinal plane (ZX) relative to the sonication path and the acoustic beam cross‐section of the focal plane (XY) are illustrated in Figure 3B,C. The lateral and vertical dimensions of acoustic beam cross‐sections measured at the full width at half (acoustic pressure) maximum were 4.58 and 4.58 mm, which makes the tFUS able to spatially target the motor cortex. The acoustic field in the axial direction, perpendicular to the transducer face, showed a peak of 28.8 mm. The spatial‐peak‐pulse‐average intensity (I_SPPA_) was 0.6156 ~ 2.4624 W/cm^2^, below the US Food and Drug Administration (FDA) recommendation of I_SPPA_ ≤ 190 W/cm^2^. The spatial‐peak time‐average intensity (I_SPTA_) was 3.078 ~ 12.312 mW/cm^2^, below the FDA recommendation of I_SPTA_ ≤ 720 mW/cm^2^ (Marketing clearance of diagnostic ultrasound systems and transducers. guidance for industry and Food and Drug Administration staff. Silver Spring, MD: US Food and Drug Administration, 2019; Figure 3D–F).

### 3.2 Effects of tFUS on motor cortex excitability

The baseline RMT and MEPs were similar across the tFUS and sham groups prior to stimulation (RMT: etFUS [38.40 ± 0.92%], itFUS [40.60 ± 1.58%], sham [39.20 ± 2.24%]; baseline MEP etFUS: [0.57 ± 0.10 mV], itFUS: [0.49 ± 0.10 mV], sham: [0.51 ± 0.11 mV]). During the study, no adverse effects were spontaneously reported or after questioning the subjects post each stimulation, supporting the safety profile of the current pulsing schemes.

**FIGURE 4:**
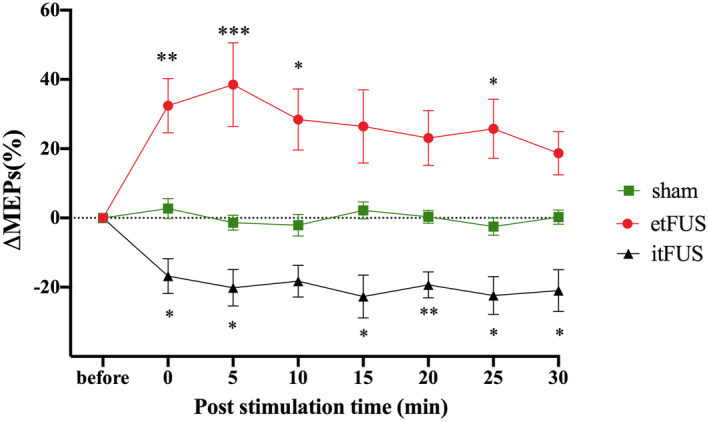
The effect of tFUS on MEPs induced by single TMS. The figure shows mean ΔMEPs percentages ± SEM. *Asterisk indicate significant difference in MEP change rate at each time point between etFUS stimulation and sham or itFUS stimulation and sham (*p < 0.05, **p < 0.01, ***p < 0.001, *n* = 10).

Two‐way rm ANOVA analysis showed significant effects of etFUS (*F*_1,18_ = 14.82, *p =* 0.0012) on the change of MEP amplitudes. Bonferroni's multiple comparisons showed that etFUS induced significant MEP increase at the time points of 0, 5, 10, and 25 min. The results indicate that etFUS increases the excitability of the motor cortex immediately after stimulation (Figure 4, Table 1).

**TABLE 1:** MEPs induced by single TMS at multiple time points.

Group	Post‐stimulation time							*F*	*p*
	0 min	5 min	10 min	15 min	20 min	25 min	30 min		
Sham	2.74 ± 2.89	−1.35 ± 2.17	−2.10 ± 3.10	2.18 ± 2.45	0.35 ± 1.84	−2.50 ± 2.49	0.24 ± 2.05		
etFUS	32.44 ± 7.84 (0.0135)*	38.49 ± 12.11 (0.0003)***	28.44 ± 8.83 (0.0101)*	26.44 ± 10.58 (0.0756)	23.09 ± 7.94 (0.1170)	25.77 ± 8.54 (0.0218)*	18.69 ± 6.23 (0.3589)	14.82	0.0012
itFUS	−16.82 ± 5.05 (0.0317)*	−20.15 ± 5.27 (0.0446)*	−18.27 ± 4.56 (0.0691)	−22.70 ± 6.18 (0.0203)*	−19.35 ± 3.75 (0.0027)**	−22.39 ± 5.46 (0.0409)*	−20.97 ± 6.08 (0.0492)*	25.04	<0.0001

*Note*: Two‐way rm ANOVA. Data are shown as mean ± SEM (*p* values of Bonferroni's multiple comparisons test compared with the sham group.

* *p* < 0.05

** *p* < 0.01

*** *p* < 0.001.

Two‐way rm ANOVA analysis showed itFUS significantly decreased MEP amplitudes (*F*_1,18_ = 25.04, *p <* 0.0001) and Bonferroni's multiple comparisons test showed the significant effects at the time points of 0, 5, 15, 20, 25, and 30 min. The results indicate that itFUS decreased the cortical excitability and the effects lasted for more than 30 min (Figure 4, Table 1).

### 3.3 Effects of tFUS on intracortical circuits

**FIGURE 5:**
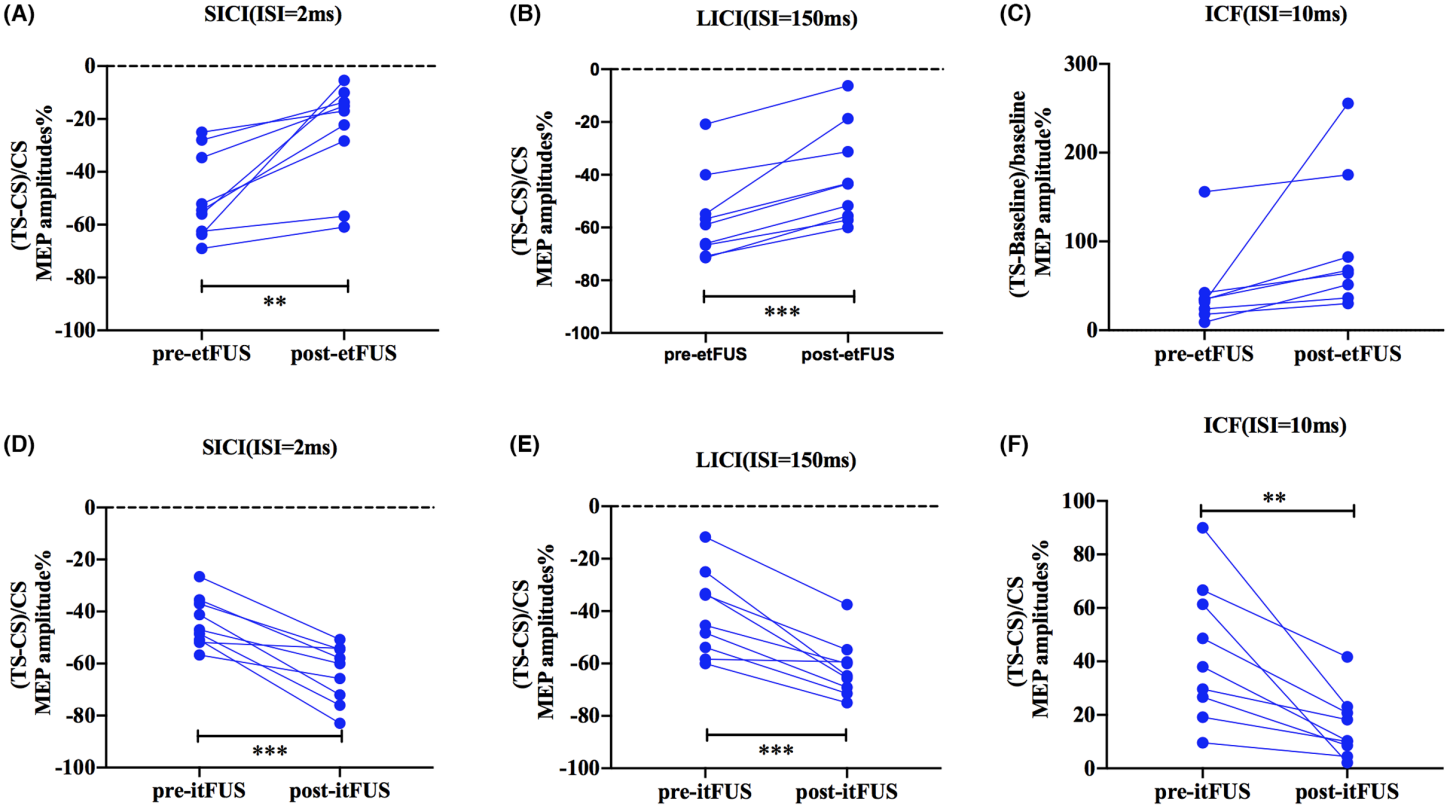
The effect of tFUS on intracortical facilitation and inhibition. Intracortical excitability profiles before and after tFUS treatment were expressed as MEP change of TS relative to the CS. SICI, LICI, and ICF before and after etFUS (A–C) and itFUS (D–F) are shown. Values of >0 indicate facilitation, and values of <0 indicate inhibition. *Asterisk significant differences between before and after tFUS stimulation (paired *t* test, *p < 0.05, **p < 0.01, *n* = 9).

The effects of tFUS on intracortical circuits reflected by SICI (ISI = 2 ms), LICI (ISI = 150 ms) and ICF (ISI = 10 ms) are shown in Figure 5. Data of ISIs at 5, 15, and 100 ms are shown in the Figure S1. Paired *t*‐test showed that the etFUS significantly reduced SICI (pre‐etFUS = −49.48 ± 5.41, post‐etFUS = −25.47 ± 6.68, *t* = 3.936, *p* < 0.01) and LICI (pre‐etFUS = −71.43 ± 5.50, post‐etFUS = −40.82 ± 6.19, *t* = 5.675, *p* < 0.001). Meanwhile, etFUS displayed a tendency to enhance ICF, but the impact is not significant (pre‐etFUS = 43.82 ± 16.46, post‐etFUS = 95.35 ± 27.90, *t* = 2.057, *p =* 0.0787). The results suggest that etFUS can mainly alleviate intracortical inhibition. Instead, itFUS significantly increased the SICI (pre‐itFUS = −43.91 ± 3.19, post‐itFUS = −63.78 ± 3.70, *t* = 5.852, *p* < 0.001) and LICI (pre‐itFUS = −41.10 ± 5.43, post‐itFUS = −61.93 ± 3.71, *t* = 5.632, *p* < 0.001) while reduced ICF (pre‐itFUS = 43.29 ± 8.58, post‐itFUS = 15.46 ± 4.05, *t* = 3.854, *p* < 0.01). The level data are given as mean ± SEM. These results suggest that itFUS can promote intracortical inhibition and suppress intracortical facilitation (Figure 5).

### 3.4 Effects of tFUS on GABA and Glx metabolites levels

**FIGURE 6:**
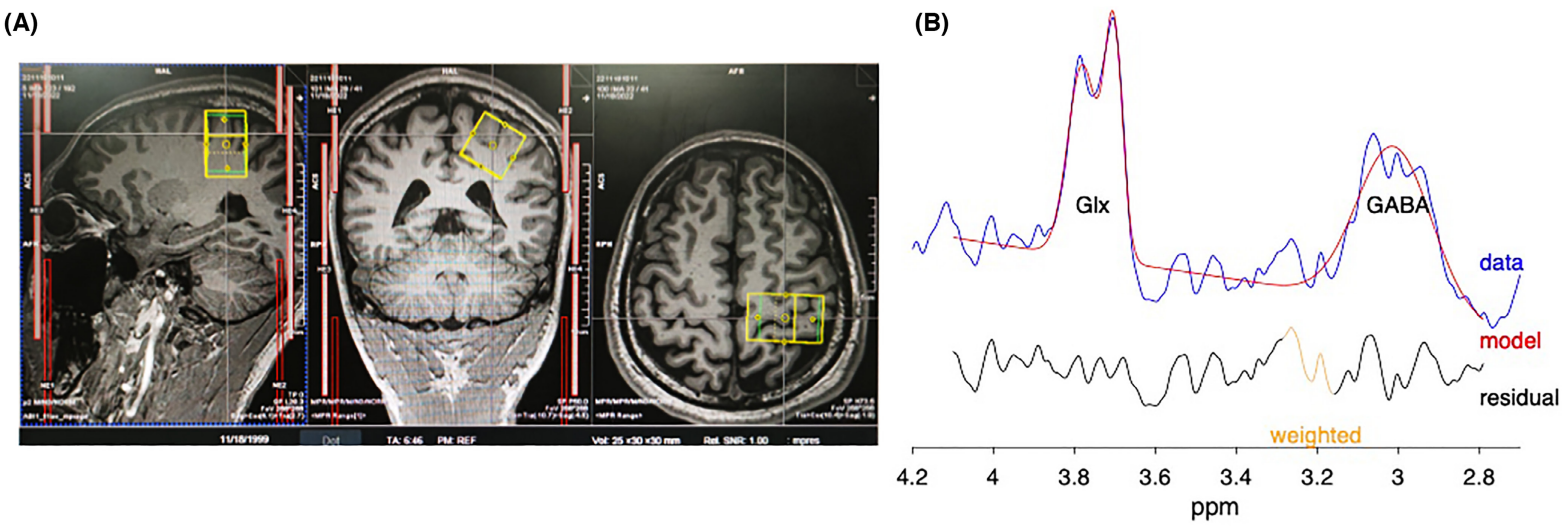
(A) Representative sagittal, coronal and axial T1‐weighted MRI brain images of a subject showing the voxel (25 × 30 × 30 mm in yellow box) within the primary hand motor cortex. (B) Curve‐fitting of the GABA and Glx peak using Gannet, the red lines in the panels are the results of the GannetFit curve‐fitting, the blue lines show the postphase and frequency aligned data, and the black line is the residual difference between the experimental data and the curve‐fit. The Glx and GABA peaks resonate at 3.7, 3.0 ppm respectively.

Edited spectra were successfully obtained from the target brain region of tFUS (Figure 6). For the etFUS, after the repeated stimulation for 7 days, the concentration of GABA decreased (6.32%), while Glx increased (12.40%), and the relative level of GABA to GLX (GABA/Glx) decreased (the paired *t* test showed that *p* = 0.095). Under the same treatment mode, the itFUS, with different parameters to etFUS, increased the GABA concentration (18.59%), decreased Glx concentration (0.35%), and there was a significantly increased GABA/Glx ratio (*p* < 0.05; Table 2).

**TABLE 2:** MRS data analysis of GABA and Glx levels.

	GABA IU (SNR)		Glx IU (SNR)		GABA/Glx	
	Pre‐treatment	Post‐treatment	Pre‐treatment	Post‐treatment	Pre‐treatment	Post‐treatment
etFUS						
Subject 1	2.440 (11.60)	2.496 (10.35)	5.229 (11.74)	6.849 (11.39)	0.467	0.364
Subject 2	3.272 (12.23)	2.778 (14.76)	7.203 (10.08)	6.820 (11.62)	0.454	0.407
Subject 3	2.702 (12.91)	2.210 (15.45)	6.168 (12.54)	7.279 (13.48)	0.438	0.304
Subject 4	2.725 (14.75)	2.881 (10.13)	6.780 (10.11)	7.181 (12.31)	0.402	0.401
Mean ± SEM	2.785 ± 0.175	2.591 ± 0.151	6.345 ± 0.428	7.032 ± 0.116	0.440 ± 0.014	0.369 ± 0.024 (*p* = 0.095)
itFUS						
Subject 5	2.911 (12.32)	4.931 (11.60)	7.578 (12.08)	8.457 (9.92)	0.384	0.583
Subject 6	2.084 (13.70)	2.266 (11.94)	6.313 (13.92)	6.190 (11.94)	0.330	0.366
Subject 7	2.135 (12.83)	2.395 (10.19)	5.103 (12.62)	5.135 (11.90)	0.419	0.466
Subject 8	2.622 (10.92)	3.216 (12.40)	6.818 (10.28)	6.633 (11.10)	0.385	0.485
Subject 9	2.907 (9.85)	2.502 (12.20)	6.658 (8.70)	5.169 (10.20)	0.437	0.384
Mean ± SEM	2.532 ± 0.180	3.062 ± 0.495	6.494 ± 0.405	6.317 ± 0.609	0.391 ± 0.018	0.477 ± 0.034*

*Note*: Significant differences of GABA/Glx between pre‐treatment and post‐treatment are tested by paired t test with *p* < 0.05 accepted as significant.

Abbreviation: SNR, Signal‐to‐Noise Ratio.

* Asterisk significant differences between before and after consecutive tFUS stimulation (*p < 0.05).

## 4 DISCUSSION

The results of this study demonstrated that precisely MRI‐guided tFUS stimulation with different parameter sets could exert excitatory and inhibitory neuromodulatory effects on human motor cortical activity. Furthermore, tFUS regulated GABAergic and glutamatergic receptor related intracortical inhibitory and excitatory processes, and altered the levels of GABA and Glx metabolites.

tFUS is a very promising new non‐invasive neuromodulation technology. It transmits acoustic mechanical vibrations to specific areas of the brain. The mechanical effects of tFUS may affect the morphology and organization of cell membranes, which in turn affects processes such as ion channel activity, synaptic protein synthesis and regulation, postsynaptic intracellular signaling, such as activation of calcium signaling pathways.^34^ To achieve clinical application, it is important to clarify the excitatory and inhibitory effects of tFUS protocol. However, the development of a reliable parameter‐dependent reference to induce excitatory and suppressive neuronal effects lies in start stage. The parameters including fundamental frequency, DC, SD and PRF determine neuromodulation effect of ultrasound. Higher fundamental frequency indicates tighter focus, more transcranial attenuation and scattering. 200–650 kHz have been used in most human and animal studies and 500 kHz is used most in human research.^13^ Plaksin et al.^35^ proposed the neuronal intramembrane cavitation excitation (NICE) model which suggests that DC determines the polarity of neuromodulation. Lower DC (i.e., below 5%) will preferentially produce inhibitory effect through activating thalamic reticular neurons (TRN), thalamocortical neurons (TCN), and low‐threshold spiking (LTS) interneurons via T‐type voltage‐gated calcium channels. Higher DC (i.e., over 20%) will preferentially produce excitatory effect through activating regular spiking (RS) pyramidal cells and fast spiking (FS) interneurons while suppressing the LTS interneurons. Yoon et al.,^36^ reported bidirectional neuromodulation effects of varying tFUS parameters, a shorter SD (≤~500 ms) at a higher DC (30%) favored excitation effect on the sensorimotor cortex and thalamus, and a longer SD (~1 min) at a lower DC (≤10%) resulted in suppression. Kim et al.^37^ found that sonication with 50% of DC outperforms 30% and 70%, pulsed tFUS outperforms equivalent continuous sonication, shorter SD (300 ms) outperforms longer SD (400 ms) stimulates the rats somatomotor more effectively. In another study, the application of pulsed 350 kHz tFUS using a 5% DC to the visual cortex area suppressed the magnitude of visual evoked potentials (VEPs) in rats while higher intensity (5 W/cm^2^) or DC (8.3%) induced slight elevation in VEPs.^38^ The study of Fomenko et al.^39^ also found a lower duty cycle (10% compared to 30% and 50%) has the greatest efficacy in suppressing motor cortex potentials. Most studies validate the proposed dependence of excitation on higher DCs predicted by the modified NICE model, while evidence for the pattern that lower DC induce inhibitory effects is insufficient.^40^ PRF may be another important parameter for the modulation of excitation‐inhibition. Yu et al.^41^ reported that the excitatory effect was more prominent at a higher PRF (3000 Hz) compared to low PRF (300 Hz) according to the increased amplitude of movement‐related cortical potential (MRCP). Badran et al.^42^ found that two 10‐min sessions of anterior thalamic tFUS (PRF = 10 Hz, DC = 5%) significantly attenuated thermal pain sensitivity in healthy individuals. In the present study, using a 500 kHz ultrasound, we found that when applied to human M1, focused ultrasound with higher DC at 40% and PRF at 2 kHz displayed excitatory effect while lower DC at 2% and PRF 50 Hz displayed inhibitory effect. MRI guidance is used to ensure the accuracy of target region individually. Although the regular pattern has not been clearly revealed, we provided possible sonication parameters for exciting or suppressing the human M1 and further explored the mechanism.

Individual features need to be considered for accurate MEP evaluation and meaningful interpretation. The study of Cantone et al.^43^ found that both MEP cortical latency and eripheral motor conduction time (PMCT) at four limbs positively correlated with age and height, while the MEP amplitude was not significantly correlated with age and height. In this study, we used MEP amplitude as an evaluation indicator, and data for each group were collected from all subjects, result biases induced by individual features can be avoided. MEP test revealed that different ultrasound stimulation parameters can cause increased or decreased cortical excitability. It is difficult to assess the exact neuronal mechanism that ultrasound affects in humans non‐invasively. We found that etFUS reduced SICI and LICI, indicating that the excitatory effect is related to the inhibition of GABA receptor function. In contrast, itFUS increased SICI and LICI while reducing ICF. Four previous studies have reported the regulatory effects of tFUS on SICI, LICI and ICF, but the results are inconsistent. The studies of Fomenko et al.^39^ reported that tFUS suppressed TMS‐elicited motor cortical activity and increased SICI, but did not significantly change LICI or ICF. Legon et al.^44^ showed that tFUS inhibited the amplitude of MEPs and attenuated ICF while did not affect SICI. Samuel et al.^45^ recently reported that a theta‐burst tFUS induced sustained increase in MEP amplitude and decreased SICI, but did not change ICF. Zeng et al.^46^ found that a similar theta‐burst tFUS produced increase in corticospinal excitability, decreased SICI, and increased ICF. The inhibitory effects and increased SICI reported by Fomenko et al. used the ultrasound parameters of fundamental frequency = 500 kHz, PRF = 1000 Hz, DC = 30%, and SD = 0.5 s. The study of Legon et al. used the same ultrasound parameters and simultaneous transcranial ultrasound and magnetic stimulation paradigm, but several methodological differences, such as time interval between ultrasonic and magnetic stimulation. These studies indicate that different parameters and experimental paradigms cause inconsistent results in intracortical circuits.

In the MRS analysis, we found that although the GABA and Glx concentrations were not significantly different, 7 days of etFUS decreased the ratio of GABA to Glx, and itFUS increased the ratio significantly. The results suggest that repeated stimulation with tFUS modulated the inhibitory and excitatory balance related to GABA and glutamate. It is believed that MRS‐GABA spectra mainly reflects extra‐synaptic concentrations that mediate tonic inhibition and regulate tonic and phasic activity in GABAergic circuits.^47^, ^48^ MRS‐Glx measures total Glx (glutamate and glutamine) concentration in a given area but cannot distinguish and quantify glutamate and glutamine reliably. It is difficult to precisely pinpoint how it relates to neurophysiological functioning. These results highlight that cortical plasticity is disturbed by tFUS. To our knowledge, this is the first study using MRS to study the role of GABA and glutamate mediated long term plasticity‐like mechanism in the ultrasonic neuromodulation.

The present study provides evidence for the sustained effect of tFUS stimulation, that is, to affect neuroplasticity by changing the receptor function and metabolic level of GABA and glutamate transmitters. The longer‐lasting effects induced by tFUS have been reported previously, e.g., reduction of SEP responses up to 35 min,^49^ reduction of fMRI BOLD response up to 2 h,^50^ and changes in connectivity.^11^, ^51^ These effects could be elicited from cortical plasticity mechanisms of long‐term potentiation (LTP) and/or depression (LTD) as proposed for other neurostimulation methods, such as transcranial direct current stimulation.^52^ Clennell et al.^53^ reported that stimulating neurons with 40 s of ultrasound enhances their excitability for up to 8 h in conjunction with modifications to action potential kinetics, suggesting the presence of plasticity‐like mechanism. Niu et al.^54^ used the long‐term potentiation/long term depression (LTP/LTD) model in the rat hippocampus, and found that tFUS at 0.5 MHz fundamental frequency for 5 min caused sustained depression of the fEPSP slope. The mechanism is unclear. tFUS's mechanical energy changes the fluidity and permeability of the membrane and induce altering conformational states and changing the capacitance of the membrane, leading to a modulation of neural activity.^55^ FUS in specific megahertz frequency bands induced microtubules vibration could stand to modulate electrical signals by influencing synaptic plasticity.^15^, ^56^ Supposedly, similar to change in membrane capacitance following repeated electrical stimulation,^57^ repeated exposure of FUS may leave lasting changes on the membrane conformational states due to stored conformation/geometric changes including ion channels.^58^ Metaplasticity refers to the modification of plasticity induction (direction, magnitude, duration) by previous activity of the same postsynaptic neuron or neuronal network.^59^ The Bienenstock–Cooper–Munro (BCM) model of homeostatic metaplasticity dictates that prior excitation will elevate the excitation threshold and thus decrease the predisposition for excitation, whereas prior inhibition will lower the excitation threshold and thus increase the predisposition for excitation.^60^ Further research on more complex metaplasticity mechanism using etFUS and itFUS priming stimulus will provide more evidence for their regulation of neuroplasticity.

There are several limitations. In experiment 1, we detected MEP within 30 min after stimulation. However, the significant effects of itFUS lasted for more than 30 min, future studies assessing long‐term temporal changes may provide greater insight into the effects of tFUS. In the mechanism study, the paired pulse TMS and MRS tests were both self‐controlled designs before and after stimulation. Since there were no multiple measurements taken in Experiment 2, the extent of LTP/LTD like plasticity remains uncertain. Our findings are limited by the relatively small sample size. The implementation of the sham control design and larger sample size will provide increased statistical capabilities. Besides, failure to measure electrophysiological indicators such as MEP alongside MRS before and after 7 consecutive days of stimulation makes it difficult to explain correlations between outcome measures.

## 5 CONCLUSION

The findings from this study demonstrate that tFUS with different parameters can exert excitatory and inhibitory neuromodulatory effects on the human motor cortex. The MEP, paired pulse TMS and MRS study revealed the neurophysiologic basis that tFUS change cortical excitability and plasticity by regulating excitatory‐inhibition balance related to the GABAergic and glutamatergic receptor function and neurotransmitter metabolic level. These findings predicted the promising application prospects of tFUS in the treatment of brain functional diseases such as psychiatric disorders, movement disorders, epilepsy, and cognitive disorders.

## AUTHOR CONTRIBUTIONS

TTZ and BQG contributed to data acquisition, data analysis, preparing the figures, and drafting the text. ZTZ contributed to the MRS data acquisition and analysis. LXJ contributed the focused ultrasound field quantification. SMH contributed to statistical analysis of data. SRL and XS contributed to data acquisition. YPW and CYL contributed to conception, study design and writing review.

## CONFLICT OF INTEREST STATEMENT

Nothing to report.

## Supporting information


Figure S1.

## Data Availability

The data that support the findings of this study are available from the corresponding author upon reasonable request.
