# Supplementary figures and images for: Excitatory‐inhibitory modulation of transcranial focus ultrasound stimulation on human motor cortex
Source: CNS Neurosci Ther. 2023 Jun 12;29(12):3829–41. doi: 10.1111/cns.14303 (PMC10651987; doi:10.1111/cns.14303)

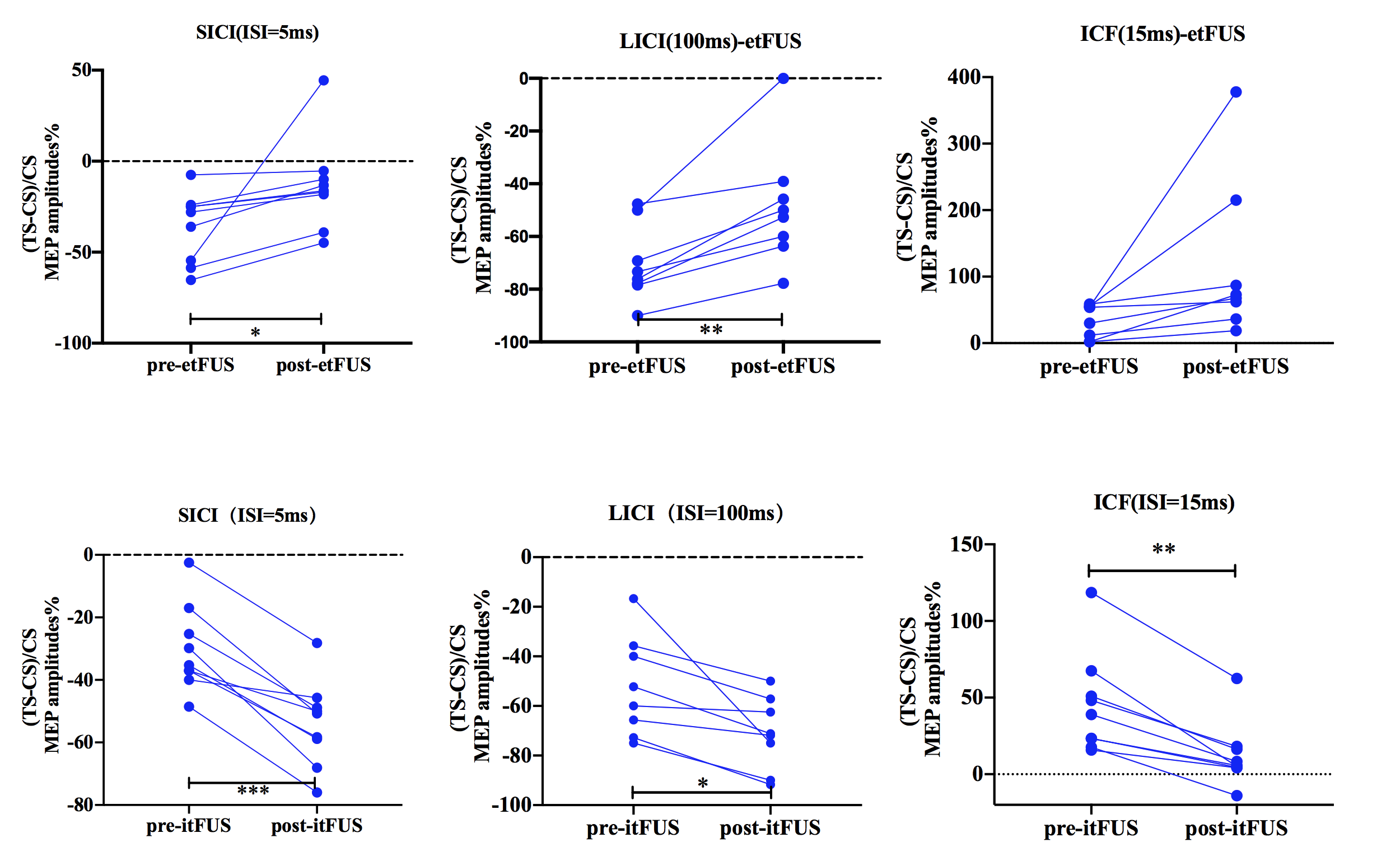

Supplement: Supplementary file 1 — Figure S1. [file CNS-29-3829-s001.tiff]
